# Risk Factors for Periprosthetic Joint Infection after Total Hip Arthroplasty and Total Knee Arthroplasty in Chinese Patients

**DOI:** 10.1371/journal.pone.0095300

**Published:** 2014-04-18

**Authors:** Chuanlong Wu, Xinhua Qu, Fengxiang Liu, Huiwu Li, Yuanqing Mao, Zhenan Zhu

**Affiliations:** Department of Orthopaedics, Shanghai Ninth People's Hospital, Shanghai Jiaotong University School of Medicine, Shanghai, People's Republic of China; University of Florida College of Medicine, United States of America

## Abstract

**Purpose:**

The purpose of this hospital-based case–control study was to evaluate the risk factors for periprosthetic joint infection (PJI) of total hip arthroplasty (THA) and total knee arthroplasty (TKA) in Chinese patients.

**Method:**

From January 2000 to December 2012, 45 patients undergoing THA and TKA who developed PJI were recruited for case subjects; controls were 252 without PJI, matched by year of index for surgery and type of surgery. Conditional logistic regressions were run to compute odds ratios (ORs) and 95% confidence intervals (CIs).

**Results:**

Demographic factors and comorbid conditions associated with an increased adjusted risk of PJI (in decreasing order of significance) were diabetes (OR = 5.47, 95% CI: 1.77–16.97; *p* = 0.003), age (65–75 vs. 45–65 years) (OR = 3.36, 95% CI: 1.30–8.69; *p* = 0.013), BMI (≥28 vs. 18.5–28 kg/m^2^) (OR = 2.77, 95% CI: 1.20–6.40; *p* = 0.017), place of residence (rural) (OR = 2.63, 95% CI: 1.13–6.10; *p* = 0.025) and alcohol abuse (OR = 2.95, 95% CI: 1.06–8.23; *p* = 0.039).

**Conclusion:**

Patients with diabetes, older age, BMI of ≥28 kg/m^2^ and alcohol abuse or living in rural areas, had increased PJI risk. Additional systematic large-scale studies are needed to verify these results.

## Introduction

The techniques of total hip arthroplasty (THA) and total knee arthroplasty (TKA) are highly successful procedures and have become conventional methods for improving quality of life and reducing pain in patients with joint diseases. Despite this, periprosthetic joint infection (PJI), a common complication of total joint arthroplasty (TJA), can have catastrophic consequences. PJI rates can reach as high as 3% following primary THA [1], and can occur in up to 2% of uncomplicated TKA cases [2].

Several demographic factors and comorbid conditions are risk factors for PJI after THA and TKA, including age [3], [4], sex [5]–[8], race [7], diabetes, obesity, alcohol abuse, preoperative diagnosis, tobacco use, congestive heart failure, chronic pulmonary disease, chronic liver disease, renal disease, preoperative anemia, oncologic disease, bone cancer, metastatic tumor, and neurologic disease [5], [6], [8]–[16]. Poultsides et al. [7] found that race was an important risk factor for PJI; compared with whites, other races had an increased risk of PJI. Namba et al. [6], [11], however, indicated that Asians (vs. whites) had no significant difference in PJI risk. To our knowledge, data on the risk factors for PJI in Asian populations is very scarce. Therefore, this study aimed to evaluate the risk factors for PJI after THA and TKA in Chinese patients.

## Methods

### Ethics statement

This case–control study is a hospital-based retrospective investigation conducted at the Shanghai Ninth People's Hospital in Shanghai, China. This study was approved by the Ethics Committee of Shanghai Ninth People's Hospital, and all participants provided written informed consent.

### Study population

From January 2000 to December 2012, patients undergoing THA or TKA who developed PJI were enrolled for cases group. Controls were patients undergoing THA or TKA without PJI, matched by year of index surgery and type of surgery. Each case was matched with 5 controls. The case and control groups did not differ significantly in terms of demographic or clinical characteristics (retrieved from patients' medical records).

### Data collection

We collected data through clinical records, including operative notes, inpatient charts, and discharge summaries. The demographic characteristics of patients included age, sex, and place of residence. To investigate the influence of age on PJI, age was stratified into 4 groups (<45 years, 45–65 years, 65–75 years, and >75 years) [7]. Place of residence was categorized as “urban” and “rural.” Because body mass index (BMI) criteria vary by country, we used the official Chinese guidelines and divided subjects into the following 2 groups: overweight (18.5–28 kg/m^2^) and obese (≥28 kg/m^2^).

The preoperative diagnosis for which THA or TKA was collected included osteoarthritis, femoral head necrosis, developmental dysplasia of the hip, fracture, rheumatoid arthritis, and other. The comorbid conditions of patients included tobacco use, alcohol abuse, diabetes, treatment of diabetes (insulin), hypertension, cardiovascular event, chronic pulmonary disease, chronic liver disease, renal disease, preoperative anemia, prostatic disorder, substance abuse, cerebral infarction, oncologic disease, neurologic disease, history of tuberculosis, gout, and ankylosing spondylitis. These comorbid conditions were chosen based on the specific diseases used to determine the composite Charlson Comorbidity Index [17] as well as other diseases used as comorbidity measures for administrative databases, which are associated with increased length of hospital stay, hospital charges, complications, and mortality [18]. Furthermore, pre-existing diseases identified as risk factors for PJI in clinical studies were also included [14]. We also collected data on dental procedures performed within 6 months before TJA. These data were collected through patients' medical records.

At the same time, we collected and reviewed previous studies examining the risk factors for PJI or surgical site infection (SSI) in patients who underwent joint arthroplasty.

### Definition of PJI

PJI in our study is present when one of the following occurs: (1) a sinus tract communicates with the prosthesis; (2) positive culture results from 2 or more tissue or fluid samples from the affected prosthesis; or (3) 4 of the following 6 criteria are present: (a) elevated serum C-reactive protein level and erythrocyte sedimentation rate, (b) elevated synovial white blood cell count, (c) high synovial polymorphonuclear (PMN) leukocyte percentage, (d) presence of purulence in the joint, (e) positive culture result from one sample from the affected joint, and (f) PMN leukocyte count of more than 5 per high-powered field in 5 high-powered fields on histologic analysis at 400× magnification.

### Statistical analysis

The distributions of demographic characteristics and comorbid conditions between cases and controls were compared using chi-squared tests. Pearson's chi-squared test was used for qualitative variable analysis, and Fisher's exact test was used when n (number of data) was less than 20 or when any value was less than 5. We systematically assessed the influence of each characteristic on the risk of PJI. All tests were 2-sided. We used conditional logistic regression to calculate odds ratios (ORs) and 95% confidence intervals (CIs) to estimate the effect of each factor on the risk of PJI. All variables were regressed on PJI with adjustment for patient characteristics. All analyses were conducted using SPSS version 18.0 (IBM Corporation, Armonk, NY) with significance set at the 5% level.

## Results

### Patient characteristics

Fifty-two cases were identified but 7 were later excluded due to incomplete data. Our final sample comprised 45 case patients (average age, 67 years; range, 41–85 years) and 252 controls (average age, 63 years; range, 24–93 years).

Table 1 presents the distributions of 45 cases and 252 controls according to age, sex, place of residence, type and diagnosis. By design, cases and controls had similar distribution by type (*p* = 0.492). Cases had a lower proportion of subjects aged 45–65 years (28.9% vs. 44.8%), living in an urban area (60.0% vs. 73.8%), and with a preoperative diagnosis of fracture (24.4% vs. 34.9%), but a higher proportion of subjects aged 65–75 years (42.2% vs. 27.0%) with a preoperative diagnosis of rheumatoid arthritis (22.2% vs. 10.7%). There was no significant difference in the distribution of sex between groups (*p* = 0.843).

**Table 1:** Patient demographics and other characteristics according to occurrence of PJI.

	Periprosthetic joint infection		
	Yes (n = 45)	No (n = 252)	P*
**Age, y**			0.107
<45	2 (4.4)	19 (7.5)	
45–65	13 (28.9)	113 (44.8)	
65–75	19 (42.2)	68 (27.0)	
≥75	11 (24.4)	52 (20.6)	
**Sex**			0.843
Male	20 (44.4)	108 (42.9)	
Female	25 (55.6)	144 (57.1)	
**Place of residence**			0.058
Rural	18 (40.0)	66 (26.2)	
Urban	27 (60.0)	186 (73.8)	
**Type**			0.492
THA	28 (62.2)	170 (67.5)	
TKA	17 (37.8)	82 (32.5)	
**Diagnosis**			0.237
Osteoarthritis	8 (17.8)	54 (21.4)	
Femoral head necrosis	8 (17.8)	30 (11.9)	
Developmental dysplasia of the hip	6 (13.3)	40 (15.9)	
Fracture	11(24.4)	88 (34.9)	
Rheumatoid arthritis	10 (22.2)	27 (10.7)	
Other	2 (4.4)	13 (5.2)	

All values are expressed as n (%).

*According to chi-square test.

Abbreviations: THA, total hip arthroplasty; TKA, total knee arthroplasty.

### Risk factors for PJI

Table 2 shows distribution of cases and controls according to patient characteristics. The case group had a higher prevalence of diabetes (37.8% vs. 9.5%, *p*<0.001), prevalence of diabetes treatment (insulin) (17.8% vs. 2.4%, *p*<0.001), prevalence of alcohol abuse (33.3% vs. 13.5%, *p* = 0.001), and proportion of patients with a BMI≥28 kg/m^2^ (57.8% vs. 36.1%, *p*<0.001). The differences of distribution according to other characteristics were not statistically significant (*p*>0.05).

**Table 2:** Patient comorbidities according to occurrence of PJI.

	Periprosthetic joint infection		
	Yes	No	P*
**Body mass index, kg/m^2^**			**<0.001**
**<18.5**	1 (2.2)	5 (2.0)	
**18.5**–**28**	18 (40.0)	156 (61.9)	
**≥28**	26 (57.8)	91 (36.1)	
**Diabetes**	17 (37.8)	24 (9.5)	**<0.001**
**Treatment of diabetes (insulin)**	8 (17.8)	6 (2.4)	**<0.001**
**Alcohol abuse**	15 (33.3)	34 (13.5)	**0.001**
Anemia	3 (6.7)	31 (12.3)	0.274
Dental procedure with antibiotic	5 (11.1)	18(7.1)	0.359
Neurologic disease	1 (2.2)	13 (5.2)	0.392
Oncologic disease	1 (2.2)	12 (4.8)	0.443
Dental procedure	8 (26.7)	35 (13.9)	0.495
Ankylosing spondylitis	1 (2.2)	11 (4.4)	0.501
Tobacco use	5 (11.1)	37 (14.7)	0.527
Hypertension	9 (20.0)	60 (23.8)	0.577
Cardiovascular event	5 (11.1)	31 (12.3)	0.822
Chronic pulmonary disease	7 (15.6)	36 (14.3)	0.824
Chronic liver disease	4 (8.9)	20 (7.9)	0.829
Prostatic disorder	4 (8.9)	25 (9.9)	0.830
Cerebral infarction	3 (6.7)	15 (6.0)	0.853
Substance abuse	4 (8.9)	24 (9.5)	0.893
Renal disease	6 (13.3)	32 (12.7)	0.907
Gout	3 (6.7)	16 (6.3)	0.936
History of tuberculosis	2 (4.4)	11 (4.4)	0.981

All values are expressed as n (%).

*According to the chi-square test.

Table 3 examines the ORs of the independent risk factors for PJI through multivariate regression analysis. Demographic factors and comorbid conditions associated with increased adjusted risk of PJI (in decreasing order of significance) were diabetes (OR = 5.47; 95% CI, 1.77–16.97; *p* = 0.003), age (65–75 years; OR = 3.36; 95% CI, 1.30–8.69; *p* = 0.013), BMI (≥28 kg/m^2^; OR = 2.77; 95% CI, 1.20–6.40; *p* = 0.017), place of residence (rural; OR = 2.63; 95% CI, 1.13–6.10; *p* = 0.025), and alcohol abuse (OR = 2.95; 95% CI, 1.06–8.23; *p* = 0.039). Dental procedures had an OR of 1.71 (95% CI, 0.38–7.70; *p* = 0.487), while dental procedures with antibiotic prophylaxis had an OR of 1.38 (95% CI, 0.20–9.54; *p* = 0.743) (Table 3). No meaningful differences were found in the other factors.

**Table 3:** Independent risk factors for PJI after multivariate regression analysis.

Patient demographics/comorbid conditions	Odds ratio	95% CI	P
**Diabetes**	5.47	1.77–16.97	**0.003**
**Age (65**–**75 y vs. 45**–**65 y)**	3.36	1.30–8.69	**0.013**
**BMI (≥28 kg/m^2^ vs. 18.5**–**28 kg/m^2^)**	2.77	1.20–6.40	**0.017**
**Place of residence (rural)**	2.63	1.13–6.10	**0.025**
**Alcohol abuse**	2.95	1.06–8.23	**0.039**
Age (>75 y vs. 45–65 y)	2.54	0.83–7.79	0.103
Treatment of diabetes (insulin)	3.69	0.63–21.69	0.148
Chronic pulmonary disease	2.35	0.68–8.15	0.177
Hypertension	0.55	0.19–1.61	0.279
Substance abuse	1.80	0.45–7.21	0.408
Cerebral infarction	0.46	0.07–3.12	0.426
Dental procedure	1.71	0.38–7.70	0.487
Renal disease	1.43	0.43–4.80	0.559
Gout	1.51	0.32–7.10	0.600
Cardiovascular event	1.32	0.39–4.52	0.654
Chronic liver disease	1.34	0.36–5.04	0.663
Anemia	0.74	0.17–3.26	0.688
Tobacco use	1.29	0.35–4.76	0.703
Ankylosing spondylitis	0.62	0.05–7.43	0.704
BMI (<18.5 kg/m^2^ vs. 18.5–28 kg/m^2^)	1.55	0.12–20.16	0.740
Dental procedure with antibiotic	1.38	0.20–9.54	0.743
THA (vs. TKA)	0.89	0.37–2.12	0.789
Male (vs. female)	0.95	0.40–2.21	0.899
Age (<45 y vs. 45–65 y)	0.91	0.15–5.40	0.919
Prostatic disorder	0.94	0.23–3.87	0.930
Oncologic disease	0.90	0.08–10.69	0.936
Neurologic disease	0.94	0.10–8.95	0.958
History of tuberculosis	1.02	0.16–6.44	0.980
**Diagnosis**			
Rheumatoid arthritis (vs. osteoarthritis)	2.81	0.69–11.47	0.151
Femoral head necrosis	2.02	0.52–7.89	0.314
Developmental dysplasia of the hip	1.54	0.40–5.89	0.527
Other	1.72	0.19–15.13	0.626
Fracture	0.83	0.24–2.89	0.772

Abbreviations: BMI, body mass index; CI, confidence interval; THA, total hip arthroplasty; TKA, total knee arthroplasty.

## Discussion

This study provides the first evidence that diabetes, age of 65–75 years, BMI≥28 kg/m^2^, rural place of residence, and alcohol abuse were independently associated with increased risk of PJI for TJA patients in a Chinese population.

### Demographic factors

#### Age

We found that patients aged 65–75 years had 3.36-fold higher risk of PJI compared with patients aged 45–65 years. Similarly, Ridgeway et al. [3] reported that patients aged 75–79 years and >80 years had a 1.56- and 1.66 -fold increased risk of PJI, respectively, compared with patients aged <65 years after all types of hip replacement. In general, older patient age would seem to coincide with poorer nutritional status and weakened immunity, resulting in elevated risk of infection.

In contrast, a single-center analysis of 8,494 joint arthroplasties, including both hip and knee procedures, showed that younger age was associated with increased risk of infection [4]. The authors explained that younger patients are generally more active than older patients; thus, younger patients potentially subject their implants to greater numbers of use cycles, leading to wear, potential revision surgery, and possibility of infection. Their study is limited, however, due to the small number of cases (43 patients). Another study[7] reported that patients aged <45 years had a 1.70-fold higher risk of PJI compared with patients aged 45–65 years. Their explanation was that this specific age group includes those undergoing TJA for inflammatory diseases (rheumatoid arthritis, systemic lupus), and those with suppressed immune systems, posttraumatic osteoarthritis after open/complex fractures necessitating multiple prior surgeries, and/or hemophilia. Other studies have reported that age had no significant effect on risk of PJI [5]–[8], [11], [19] (**Table 4** **and** **Table 5**). More trials are needed to research the mechanism between age and PJI.

**Table 4:** Data from previous studies examining risk factors for periprosthetic joint infection (PJI) or surgical site infection (SSI) in patients who underwent THA and TKA.

Author	Year	Country	Research type	Total number of patients	Position	Statistical method
Bozic et al.	2013	USA	Case–control	587	THA-PJI	Multivariate Cox regression
Namba et al.	2013	USA	Cohort study	56,216	TKA-SSI	Multivariate Cox regression
Everhart at al.	2013	USA	Cohort study	1,891	TJA-SSI	Logistic regression
Poultsides et al.	2012	USA	Cohort study	1,196,691	Hip and knee arthroplasty-PJI	Logistic regression
Namba et al.	2012	USA	Cohort study	30,491	THA-SSI	Multivariate Cox regression
Dale et al.	2012	Norway	Cohort study	432,168	THA-PJI	Multivariate Cox regression
Bozic et al.	2012	USA	Cohort study	40,919	THA-PJI	Multivariate Cox regression
Bozic et al.	2012	USA	Cohort study	83,011	TKA-PJI	Multivariate Cox regression
Song et al.	2012	Republic of Korea	Cohort study	6,848	THA,TKA-SSI	Logistic regression
Peel et al.	2011	Australia	Case–control	189	Hip and knee arthroplasty-PJI	Logistic regression
Pulido et al.	2008	USA	Cohort study	9,245	Hip and knee arthroplasty-PJI	Logistic regression
Babkin et al.	2007	Israel	Case-control	180	TKA-SSI	Logistic regression
Kasteren et al.	2007	Holland	Cohort study	1,922	THA-SSI	Logistic regression
Lai et al.	2007	Canada	Case-control	52	Hip and knee arthroplasty-PJI	Logistic regression
Ridgeway et al.	2005	UK	Cohort study	24,808	Hip arthroplasty-SSI	Logistic regression

Abbreviations: THA, total hip arthroplasty; TKA, total knee arthroplasty; TJA, total joint arthroplasty.

**Table 5:** Data from previous studies examining risk factors (positive results in this article) for periprosthetic joint infection or surgical site infection in patients who underwent THA and TKA, in detail.

Literatures	Patient demographics/comorbid conditions	Age, y	BMI, kg/m^2^	Diabetes	Alcohol abuse
**Bozic et al. (2013)**	**HR (95% CI), P-value**	**≤44 vs. >75:** 0.47 (0.13–1.70), 0.25; **45**–**54 vs. >75**: 1.01 (0.43–2.36), 0.99; **55**–**64 vs. >75**: 1.22 (0.59–2.54), 0.59; **65**–**74 vs. >75**: 0.90 (0.40–2.00), 0.80	**Obesity**: 2.12 (1.08–4.16), 0.03	1.11 (0.53–2.30), 0.79	
**Namba et al. (2013)**	**HR (95% CI), P-value**	**1-year increment**: 0.99 (0.98–1.00), 0.17	**≥35 vs. <35**: 1.47 (1.17–1.85), 0.01	1.28 (1.03–1.60), 0.03	
**Everhart et al. (2013)**	**OR (95% CI), P-value**		**≥50**: 5.28 (1.38–17.1), 0.01	1.83 (1.02–3.27), 0.05	
**Poultsides et al. (2013)**	**OR (95% CI), P-value**	**0**–**44 vs. 45**–**64**: 1.70 (1.48–1.95), 0.01; **65**–**74 vs. 45**–**64**: 0.90 (0.83–0.97), 0.01; **≥75 vs. 45**–**64**: 0.98 (0.90–1.06), 0.23	**Obesity**: 0.87 (0.78–0.97), 0.01	**Uncomplicated diabetes**: 0.95 (0.86–1.04), 0.28 **Complicated diabetes**: 1.54 (1.22–1.94), 0.01	1.57 (1.23–2.00), 0.01
**Namba et al. (2012)**	**HR (95% CI), P-value**	**1-year increment**: 1.00 (0.98–1.01), 0.72	**<18.5 vs. 18.5**–**30**: 2.88 (0.89–9.23), 0.08; **30**–**35 vs. 18.5**–**30**: 1.56 (1.03–2.37), 0.04; **≥35 vs. 18.5**–**30**: 2.37 (1.55–3.61), <0.01	1.25 (0.85–1.83), 0.26	
**Dale et al. (2012)**	**HR (95% CI), P-value**	**40**–**51 vs. <40**: 1.1 (0.8–1.5), 0.6; **60**–**69 vs. <40**: 1.1 (0.8–1.5), 0.7; **70**–**79 vs. <40**: 1.1 (0.8–1.5),0.6; **80**–**89 vs. <40**: 0.9 (0.7–1.3), 0.8;**≥90 vs. <40**: 0.7 (0.4–1.4), 0.3			
**Bozic et al. –THA(2012)**	**HR (95% CI), P-value**		**Obesity**: 1.73 (1.35–2.22), 0.01	1.31 (1.12–1.53), 0.06	1.72 (0.98–3.01), 0.06
**Bozic et al.–TKA (2012)**	**HR (95% CI), P-value**		**Obesity**: 1.22 (1.03–1.44), 0.02	1.19 (1.06–1.34), 0.01	1.11 (0.63–1.97), 0.71
**Song et al. (2012)**	**OR (95% CI), P-value**			**TKA and THA**: 1.56 (1.10–2.21), >0.05 **THA**: 1.97 (1.20–3.22), >0.05	
**Peel et al. (2011)**	**OR (95% CI), P-value**			**Knee replacement**: 1.5 (0.9–2.7), 0.2; **Hip replacement**: 1.4 (0.8–2.2), 0.2; **Hip and knee replacements**: 1.4 (0.9–2.1), 0.06	
**Pulido et al. (2008)**	**OR (95% CI), P-value**		**≥40**: 3.23 (1.6–6.5), 0.01		
**Kasteren et al. (2007)**	**OR (95% CI), P-value**	**Per 10-year increase**: 1.4 (1.0–2.1), 0.08			
**Lai et al. (2007)**	**OR (95% CI), P-value**		**Obesity (BMI>30)**: NS	3.91 (1.06–14.44), 0.04	
**Ridgeway et al. (2005)**	**OR (95% CI), P-value**	**65**–**74 vs. <65**: 1.13 (0.85–1.50), <0.01; **75**–**79 vs.** <65: 1.56 (1.16–2.10), <0.01; **≥80 vs. <65**: 1.66 (1.24–2.21), <0.01			

Abbreviations: BMI, body mass index; HR, hazard ratio; NS, not significant; OA, osteoarthritis; OR, odds ratio; THA, total hip arthroplasty; TKA, total knee arthroplasty; RR, relative risk.

#### Place of residence

To our knowledge, no study has specifically reported place of residence to be a risk factor associated with incidence of PJI. We found that compared with urban patients, those living in rural areas had a 2.63-fold increased risk of PJI. This may be due to more activities, delay in diagnosing the underlying disease, irregular treatment, or difficulty paying medical expenses in some rural areas. The difference between city and rural areas also may be explained in terms of social status and education. The specific reasons for increased risk in patients living in rural require further elucidation.

### Comorbid conditions

#### Diabetes

In the current study, patients with diabetes had an increased risk of PJI (adjusted OR = 5.47; 95% CI, 1.77–16.97) compared with patients without diabetes. Similarly, a case–control study focusing on primary hip and knee arthroplasties, conducted by Lai et al. [12], showed that PJI incidence was 3.91 times higher in patients reporting a history of diabetes. Jamsen et al. [13] analyzed the one-year incidence of PJI in a single-center series of 7,181 patients with primary hip and knee replacements, conducted between 2002 and 2008, and found that diabetes more than doubled the risk of PJI, independent of obesity (adjusted OR = 2.3; 95% CI, 1.1–4.7). Several studies have also suggested that diabetes increases the risk of postoperative infection in TJA patients [6], [7], [9], [10] (**Table 4** **and** **Table 5**).

Previous literature has suggested several mechanisms behind the relationship between diabetes and PJI, but no definite conclusions have been made. On one hand, Joshi et al. reported that diabetes seems to increase the likelihood of infection caused by certain bacteria, such as *Staphylococcus aureus* [20], which is a susceptible bacteria for PJI. On the other hand, whether adaptive immune responses are affected by diabetes is still controversial [21], [22]. Finally, multiple factors have been cited as affecting the risk of PJI in diabetic patients, including type of hyperglycemia treatment, glycemic control, occurrence of diabetes-related complications, and postoperative hyperglycemia [23]–[26]. In contrast, some studies have reported that diabetes has no effect on PJI risk [5], [15], [27], [28] (**Table 4** **and** **Table 5**). In summary, more research, particularly looking at the molecular mechanisms of diabetes affecting PJI risk, is urgently needed.

#### BMI

Our data showed that patients with a BMI greater than 28 kg/m^2^ had a 2.77 -fold higher risk of PJI compared with patients with a BMI between 18.5 and 28 kg/m^2^. Likewise, in 2013, Bozic et al. [5] found that obesity independently increased the risk of PJI by 2.12 times after reviewing 587 patients who underwent THA. In another study examining the Medicare sample claims database of 40,919 patients who underwent primary THA, obesity was associated with a 1.73 -fold increased risk of PJI [15]. Several other studies also support our results regarding the effect of BMI on PJI risk [6], [9], [11], [14], [15]. Reduced immunity and increased susceptibility of obese individuals to infection may explain why obese patients more easily develop PJI [29]. In contrast, Poultsides et al. [7] indicated that obesity was associated with a 0.87-times-fold decreased risk of PJI in patients who underwent primary hip and knee arthroplasties. The authors explained that the reason for this may be that readmission data were not captured and these patients may present more commonly after discharge from the hospital, especially since the length of stay is now 3–4 days (or less) for most TJAs.

#### Alcohol

Our data showed alcohol abuse to carry a 2.95-fold higher risk of PJI. Very few studies have found alcohol abuse to be associated with an increased risk of infection in TJA patients. In a 10-year study of patients who underwent primary hip and knee arthroplasties, Poultsides et al. [7] found that alcohol abuse carried a 1.57-fold higher risk of PJI. Excessive alcohol consumption plays an important role in the development of cirrhosis, which shows an increased incidence of bacterial infection [30]. Impaired phagocytic function may be another important reason [31]. In addition, studies have shown that alcohol affects both branches of the immune system on multiple levels and disrupts many immune mechanisms [32].

#### Dental procedures

Bacteremia from distant infectious foci may cause prosthesis contamination through a hematogenic route. Dental is one of the primary foci most frequently reported in the worldwide literature. The association of dental procedures with risk of PJI has been actively debated for many years. Compared with dental procedure without antibiotic prophylaxis, we found that dental procedure with antibiotic prophylaxis had a lower risk of PJI, but that both dental procedure with or without antibiotic prophylaxis were not significant risk factors for PJI (*p*>0.05). The new “American Academy of Orthopedic Surgeons (AAOS) and American Dental Association (ADA) Clinical Practice Guideline on Prevention of Orthopaedic Implant Infection in Patients Undergoing Dental Procedures” [33] indicates that dental procedures are not risk factors for subsequent total hip or knee infection. The use of antibiotic prophylaxis prior to dental procedures did not decrease the risk of subsequent total hip or knee infection [34]. Our results support the guideline.

Previous studies have found that TKA (vs. THA) [14], male (vs. female) sex [5]–[8], femoral head necrosis (vs. osteoarthritis) [6], [8], rheumatic disease [10], [15], hip fracture [8], posttraumatic arthritis [6], inflammatory disease [8], tobacco use [9], congestive heart failure [7], [10], chronic pulmonary disease [7], [10], chronic liver disease [7], renal disease [7], [10], preoperative anemia [10], [15], oncologic disease [7], bone cancer [9], metastatic tumor [10], and neurologic disease [7] are significant increased risk factors for PJI, and that male (vs. female) sex [11] significantly decreases the risk of PJI (**Table S1**). However, we did not find these conditions to be significantly associated with PJI risk after controlling for all clinical and demographic factors in our study (*p*>0.05). Different circumstances, races, study designs, and statistical analyses may explain the discrepancies between various studies. Our findings are of significant importance to Chinese individuals.

### Strengths and limitations of this study

Our study has several strengths over previous studies on this topic. First, to the best of our knowledge, this is the first case–control study to investigate the effect of patient factors on PJI risk in a Chinese population. Compared with whites, Chinese individuals use more antibiotics when they have a common cold or other diseases. Therefore, we believe it is very significant to investigate the risk factors for infection in the Chinese population. Second, we found that patients living in rural areas had an increased risk of PJI compared with urban patients. This is a warning for rural patients and local government. Third, we confirmed the point of the new “AAOS-ADA Clinical Practice Guideline on Prevention of Orthopaedic Implant Infection in Patients Undergoing Dental Procedures.”

Nevertheless, this study has some limitations. First, more than 90% of individuals with diabetes had the type 2 variant, which is believed to have multiple causes (including obesity). We were unable to discriminate between type 1 and type 2 diabetes in our analyses; hence, we could not analyze the association between diabetes subtype and PJI risk. Second, the number of patients with PJI was rather low, which may have reduced the generalizability of our conclusions. Finally, because of the low number of study subjects, we did not distinguish between high-risk and low-risk dental procedures. This may have affected the accuracy of the influence of dental procedure on PJI risk.

### Conclusion

In conclusion, patients with diabetes, age of 65–75 years, BMI≥28 kg/m^2^, alcohol abuse, or residence in rural areas had increased risk of PJI in this Chinese population. Further systematic studies evaluating the risk factors for PJI in Asian populations are needed to confirm these findings.

## Supporting Information

Table S1 **Data from previous studies examining risk factors(others) for periprosthetic joint infection or surgical site infection in patients who underwent THA and TKA, in detail.**
(DOC)
