# Supplementary material for: Risk Factors for Periprosthetic Joint Infection after Total Hip Arthroplasty and Total Knee Arthroplasty in Chinese Patients
Source: PLoS One. 2014 Apr 18;9(4):e95300. doi: 10.1371/journal.pone.0095300 (PMC3991645; doi:10.1371/journal.pone.0095300)
Supplement: Table S1 — Data from previous studies examining risk factors(others) for periprosthetic joint infection or surgical site infection in patients who underwent THA and TKA, in detail. (DOC) [file pone.0095300.s001.doc]

Table S1.Data from previous studies examining risk factors(others) for periprosthetic joint infection or surgical site infection in patients who underwent THA and TKA, in detail

|  | **Bozic et al.(2013)** | **Namba et al.(2013)** | **Everhart et al. (2013)** | **Poultsides et al.(2013)** | **Namba et al.(2012)** | **Dale et al.(2012)** | **Bozic et al. –THA(2012)** |
| --- | --- | --- | --- | --- | --- | --- | --- |
| **Patient demographics/ comorbid conditions** | **HR (95%CI),**  **P-value** | **HR (95%CI),**  **P-value** | **OR (95%CI),**  **P-value** | **OR(95% CI),**  **P-value** | **HR (95%CI),**  **P-value** | **HR (95%CI),**  **P-value** | **HR (95%CI),**  **P-value** |
| **Sex** | **Female vs.male:**  0.55 (0.30–0.99), 0.05 | **Male vs. female:**  1.89 (1.54–2.32), <0.01 |  | **Male vs.female:**  1.33 (1.25–1.42),0.01 | **Male vs. female:**  0.70 (0.49–0.99), 0.04 | **Male vs. female:**  1.9 (1.8–2.1), <0.01 |  |
| **THA vs. TKA** |  |  |  | NS |  |  |  |
| **Diagnosis** |  |  |  |  |  |  |  |
| **Femoral head necrosis vs. OA** |  | 3.65 (1.41–9.46), 0.01 |  |  | NS | 1.7 (1.4–2.1), <0.01 |  |
| **Developmental dysplasia of the hip** |  |  |  |  |  | **Childhood hip disease:**  0.9 (0.7–1.2), 0.6 |  |
| **Fracture vs. OA** |  |  |  |  |  | 2.1 (1.9–2.4), <0.01 |  |
| **Rheumatoid arthritis vs. OA** | **Rheumatic disease:**  NS |  |  |  | NS |  | **Rheumatic disease:**  1.71 (1.42–2.06), <0.01 |
| **Other vs. OA** |  | **Posttraumatic arthritis:**  3.23 (1.68–6.23), <0.01 |  |  |  | **Inflammatory disease:**  1.4 (1.1–1.7), 0.01 |  |
| **Preoperative anemia** |  |  |  |  |  |  | 1.36 (1.15–1.62), 0.04 |
| **Neurologic disease** |  |  |  | 1.29 (1.08–1.54), 0.01 |  |  |  |
| **Oncologic disease** | **Malignancy:**  NS |  | **Bone cancer:**  3.86 (1.21–12.79), 0.04 | 1.99 (1.69–2.35), 0.01 |  |  | **Metastatic tumor:** NS;  **Malignancy:**  NS |
| **Tobacco use** |  |  | 2.96(1.65–5.11), 0.01 |  |  |  |  |
| **Hypertension** | NS |  |  |  |  |  | NS |
| **Cardiovascular event** | **Ischemic heart disease:**  NS |  |  | **Congestive heart failure:**  2.10 (1.85–2.38), 0.01 |  |  | **Congestive heart failure:**  NS  **Ischemic heart disease:**  NS |
| **Chronic pulmonary disease** | NS |  |  | 1.43 (1.32–1.55), 0.01 |  |  | NS |
| **Chronic liver disease** | NS |  |  | 2.53 (2.01–3.19), 0.01 |  |  | NS |
| **Cerebral infarction** |  |  |  |  |  |  | NS |
| **Substance abuse** | NS |  |  |  |  |  | NS |
| **Renal disease** | NS |  |  | 1.75 (1.45–2.10), 0.01 |  |  | NS |

Table S1. continued

|  | **Bozic et al.–TKA(2012)** | **Song et al.(2012)** | **Peel et al.(2011)** | **Pulido et al.(2008)** | **Babkin et al.(2007)** | **Kasteren et al.(2007)** | **Lai et al.(2007)** |
| --- | --- | --- | --- | --- | --- | --- | --- |
| **Patient demographics/ comorbid conditions** | **HR (95%CI),**  **P-value** | **OR (95%CI),**  **P-value** | **OR (95%CI),**  **P-value** | **OR (95%CI),**  **P-value** | **RR (95%CI),**  **P-value** | **OR (95%CI),**  **P-value** | **OR (95%CI),**  **P-value** |
| **Sex** |  | **TKA:Male vs.female:**  2.84 (1.71–4.74),  >0.05 |  |  | **Male vs.female:** NS | **Female vs.male:**  NS |  |
| **THA vs. TKA** |  |  |  | 2.85 (1.5–5.6), 0.01 |  |  |  |
| **Diagnosis** |  |  |  |  |  |  |  |
| **Femoral head necrosis vs. OA** |  |  |  |  |  |  |  |
| **Developmental dysplasia of the hip** |  |  |  |  |  |  |  |
| **Fracture vs. OA** |  |  |  |  |  |  |  |
| **Rheumatoid arthritis vs. OA** | **Rheumatic disease:**  1.18 (1.02–1.37), 0.03 |  | NS |  |  |  | NS |
| **Other vs. OA** |  |  |  |  |  |  |  |
| **Preoperative anemia** | 1.26 (1.09–1.45), 0.01 |  |  |  |  |  |  |
| **Neurologic disease** |  |  |  |  |  |  | NS |
| **Oncologic disease** | **Metastatic tumor:**  1.59 (1.03–2.47), 0.04;  **Malignancy:**  NS |  |  |  |  |  |  |
| **Tobacco use** |  |  |  |  |  |  |  |
| **Hypertension** | NS |  |  |  |  |  |  |
| **Cardiovascular event** | **Congestive heart failure:**  1.28 (1.13–1.46), <0.01;  **Ischemic heart disease:**NS |  |  |  |  |  | NS |
| **Chronic pulmonary disease** | 1.22 (1.10–1.36), <0.01 |  |  |  |  |  | NS |
| **Chronic liver disease** | NS |  |  |  |  |  |  |
| **Cerebral infarction** | NS |  |  |  |  |  |  |
| **Substance abuse** | NS |  |  |  |  |  | **Chronic steroid use:** NS |
| **Renal disease** | 1.38 (1.11–1.71), 0.01 |  | NS |  |  |  | NS |

Abbreviations: BMI, body mass index; HR, hazard ratio; NS, not significant; OA, osteoarthritis; OR, odds ratio; THA, total hip arthroplasty; TKA, total knee arthroplasty; RR, relative risk.
